# Hyperparathyroidism-Jaw Tumor Syndrome: A Case Diagnosed After the Removal of Multiple Ossifying Fibromas of the Jaws

**DOI:** 10.1210/jcemcr/luaf049

**Published:** 2025-03-20

**Authors:** Pål Steinmo Johnsen, Martin Madsen, Anja Nilsen Nyland, Khalid Al-Shibli, Paula Frid

**Affiliations:** Department of Otorhinolaryngology, Division of Oral and Maxillofacial Surgery, Nordland Hospital Trust, Bodø 8092, Norway; Department of Clinical Dentistry, UiT the Arctic University of Norway, Tromsø 9019, Norway; Department of Otorhinolaryngology, Division of Oral and Maxillofacial Surgery, Nordland Hospital Trust, Bodø 8092, Norway; Department of Clinical Dentistry, UiT the Arctic University of Norway, Tromsø 9019, Norway; Department of Otorhinolaryngology, Division of Oral and Maxillofacial Surgery, Nordland Hospital Trust, Bodø 8092, Norway; Department of Pathology, Nordland Hospital Trust, Bodø 8092, Norway; Department of Clinical Dentistry, UiT the Arctic University of Norway, Tromsø 9019, Norway; Department of Otorhinolaryngology, Division of Oral and Maxillofacial Surgery, University Hospital North Norway, Tromsø 9038, Norway; Public Dental Service Competence Centre of North Norway, Tromsø 9019, Norway

**Keywords:** hyperparathyroidism jaw tumor syndrome, ossifying fibroma, *CDC73*, parathyroid carcinoma

## Abstract

Hyperparathyroidism-jaw tumor syndrome (HPT-JT) is a hereditary neoplastic disorder caused by a pathogenic variant in the *CDC73* tumor suppressor gene. It is characterized by increased values of PTH, ossifying fibromas of the jaws, and in some cases neoplasms of the kidneys/or the uterus. We present a case of a 65-year-old male who had several jaw tumors with secondary infection requiring treatment. Previous medical history included parathyroid adenomas with a long history of increased PTH and kidney tumor. This prompted genetic testing, which confirmed a heterozygous *CDC73* pathogenic variant, establishing the diagnosis of HPT-JT. The jaw tumor was excised. The patient was referred to genetic counselling but declined.

## Introduction

Hyperparathyroidism-jaw tumor syndrome (HPT-JT) is a rare autosomal dominant endocrine neoplastic disorder characterized by increased values of PTH due to neoplasms of parathyroid glands, in 10% to 50% of cases ossifying fibromas of the jaws and in 15% neoplasms of the kidneys and/or the uterus [1, 2].

The responsible gene was identified in 2002 as cell division cycle 73 (*CDC73*), a tumor suppressor gene located to chromosomal region 1q31.2 encoding the protein parafibromin. Inactivation of this gene and its loss of parafibromin expression is directly involved in predisposition to HPT-JT [3]. Primary hyperparathyroidism (PHPT) is a more common endocrine disease in which there is an increase of PTH. Clinical manifestation of severe PHPT can lead to high bone turnover with occurrence of osteoclastomas—so called “brown tumors.” The fibro-osseous jaw tumors of HPT-JT are distinct from brown tumor, which can occur with PHPT [4]. The ossifying fibromas of HPT-JT are benign but can be locally aggressive, causing functional and cosmetic problems if not treated. They are thought to arise from the periodontal ligament [5].

Parathyroid carcinoma (PC) is exceedingly rare, accounting for 0.005% of all cancers. HPT-JT is also related to an extreme increased risk for parathyroid carcinoma, and a 15% to 31% occurrence in patients with HPT-JT has been reported. The pathogenic variant of *CDC73* has been found to be a major driver in the etiology of PC [2, 6]. The prevalence of HPT-JT is unknown [7].

## Case Presentation

**Figure 1.**
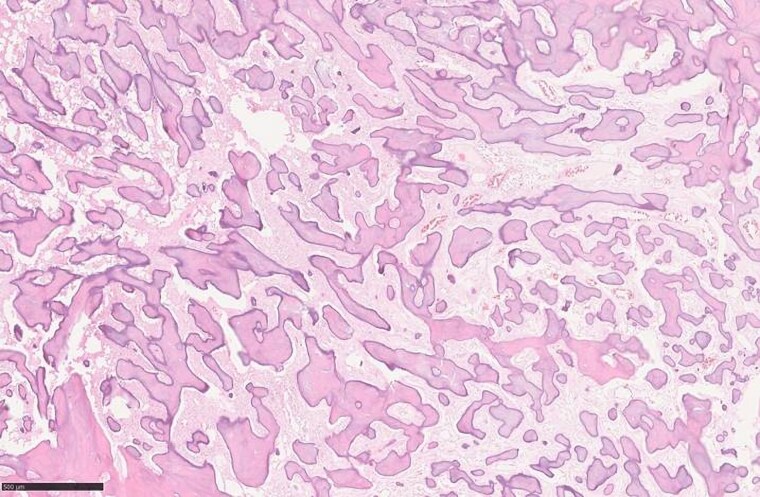
Histopathological slide at 100 × magnification, hematoxylin and eosin stain, of the intraosseous lesion biopsied in 2017 reveals areas of benign fibrous stroma with islands of immature bone. The slide demonstrates abnormal deposition of irregularly oriented and partially immature lamellar bone surrounded by fibrous stroma containing uniform fibroblasts. Diagnosis: ossifying fibroma. Image courtesy of Department of Pathology, Nordland Hospital Trust.

A 65-year-old male patient from Norway presented with an extraoral swelling and fistula at the base of the right mandible. A firm submandibular lump that seemed to be attached to the mandible could be palpated extraorally. The patient reported no pain from this area. Oral examination revealed an edentulous region in the right lower jaw with an intraoral fistula, where on palpation pus was extruded. There was no palpable intraoral expansion of the jawbone. The patient's medical history included a psychiatric disorder, chronic kidney disease, and Wilms tumor, which was treated with nephrectomy at age 4. Earlier medical records indicated increased PTH and initially diagnosed as tertiary hyperparathyroidism. This was later revised to primary hyperparathyroidism after localization of an uniglandular parathyroid adenoma, which was surgically removed in 1983. An additional adenoma was located by scintigraphy in 2002 and managed with observation. Elevated PTH levels were first noted in 1994, and the patient has been monitored with annual biochemical testing by a nephrologist since 1999. These levels, along with serum calcium and total vitamin D, are presented in Table 1. A fibro-osseous lesion of the upper left maxilla had been monitored since 1994, removed in 2017 due to infection, and histologically diagnosed as ossifying fibroma (Fig. 1).

**Table 1.** Summary of biochemical test of patient presenting with hyperparathyroidism-jaw tumor syndrome (1994-2023)

Biochemical test	Time period	Measurement range	Mean ± SD	Normal reference range
PTH	1994-2023	103.7-537.5 pg/mL (11-57 pmol/L)	263.1 ± 88.4 pg/mL (27.9 ± 9.37 pmol/L)	12.3-80.2 pg/mL (1.3-8.5 pmol/L)
Total calcium	1999-2023	8.34-11.82 mg/dL (2.08-2.95 mmol/L)	9.74 ± 0.56 mg/dL (2.43 ± 0.14 mmol/L)	8.62-10.06 mg/dL (2.15-2.51 mmol/L)
Total vitamin D	2010-2023	13.2-28.0 ng/mL (33-70 nmol/L)	22.56 ± 4.328 ng/mL (56.38 ± 10.82 nmol/L)	20-60 ng/mL (50-150 nmol/L)

Values in parentheses are International System of Units.

## Diagnostic Assessment

**Figure 2.**
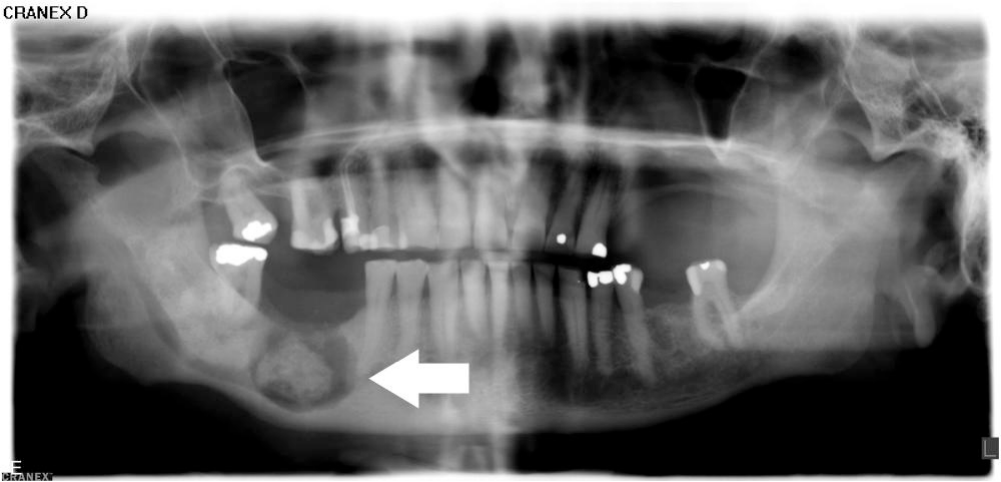
Panoramic x-ray of the jaws from 2023 showing an intraosseous lesion in the lower right mandible.

**Figure 3.**
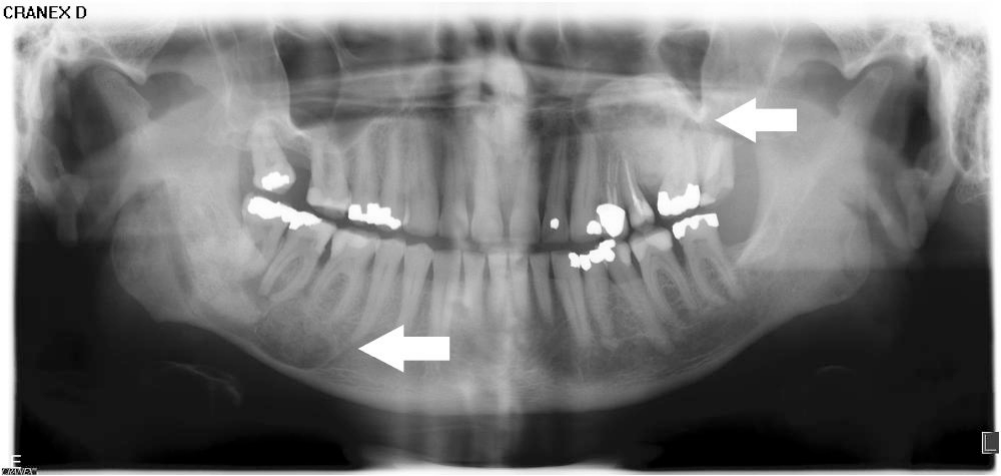
Panoramic x-ray of the jaws from 2014 showing an intraosseous lesion in the lower right mandible and upper left maxilla.

**Figure 4.**
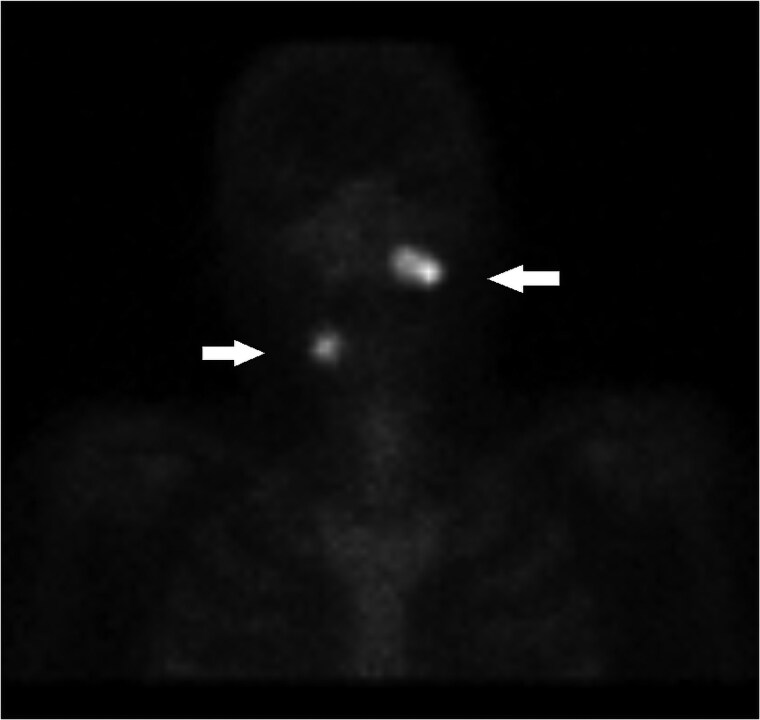
Scintigraphy with Technetium-99 m from 2007 revealed hypermetabolic areas in the right mandible and left maxilla concordant with a later diagnosis of osseous fibromas. Image courtesy of the Department of Radiology, Nordland Hospital Trust.

**Figure 5.**
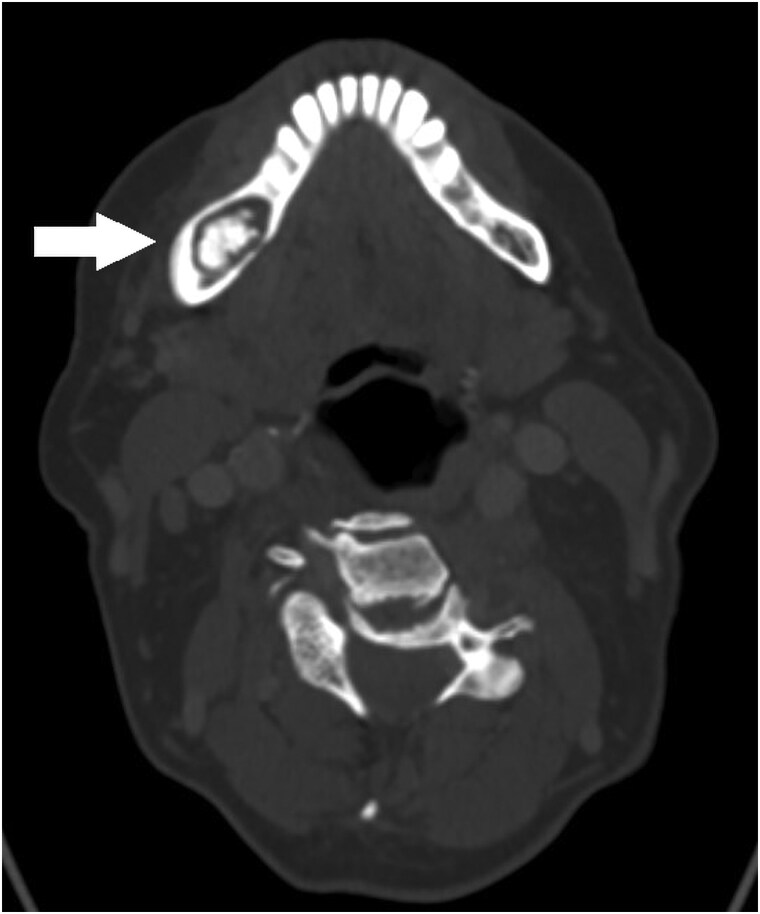
Computed tomography scan (axial view) from 2023 showing an intraosseous lesion of the right mandible. Image courtesy of the Department of Radiology, Nordland Hospital Trust.

**Figure 6.**
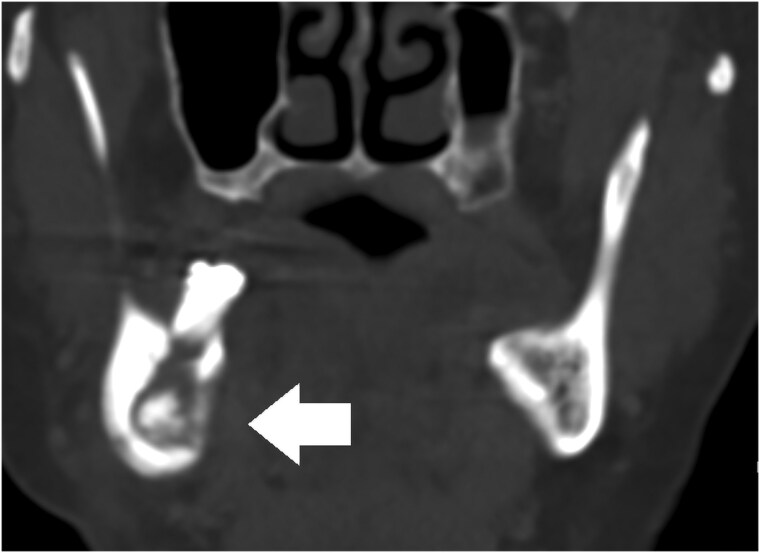
Computed tomography scan (coronal view) from 2023 showing an intraosseous lesion of the right mandible. Image courtesy of the Department of Radiology, Nordland Hospital Trust.

Diagnostic studies included a panoramic x-ray, a computed tomography-scan (CT), blood works, ultrasonography of neck, biopsy of jaw lesion, and genetic testing. The panoramic x-ray revealed a sequestered lesion in the lower right mandible (Fig. 2). Earlier panoramic x-rays showed a previously suspected fibro-osseous lesion located in the same area (Fig. 3). The surrounding teeth had been removed in 2020. Scintigraphy with Technetium-99 m from 2007 had revealed hypermetabolic areas in right mandible and left maxilla but was interpreted by the radiologist as odontogenic infection (Fig. 4). CT was examined by a radiologist who indicated ossifying fibroma as the radiographic diagnosis and advised further testing for HPT-JT (Figs. 5 and 6). Biochemical test results revealed normocalcemia (2.29 mmol/L; 9.18 mg/dL, normal reference range: 2.11-2.52 mmol/L; 8.46 mg/dL-10.1 mg/dL) and elevated PTH levels (29 pmol/L; 273.47 pg/mL, normal reference range 1.3-8.5 pmol/L; 12.26-80.16 pg/mL). Genetic testing for HPT-JT was performed at Oslo University Hospital, revealing a heterozygous *CDC73* pathogenic variant.

## Treatment

The patient underwent removal of sequestrum with debridement and securing of histological and microbial samples. Intravenous antibiotics penicillin (1.2 g, 4 times daily) and metronidazole (1 g, once daily) were initiated perioperatively after securing microbial samples. Healing was uncomplicated.

Biopsy showed only necrotic bone, and microbiological samples from mandibular bone marrow and deep sequestrum revealed a polymicrobial flora. Bloodwork revealed no increased C-reactive protein or leukocytosis.

## Outcome and Follow-up

A 1-year follow-up of the jaws with panoramic x-ray revealed no new jaw tumors. The patient was referred to the Department of Medical Genetics at University Hospital of North Norway for genetic counseling, but he declined further counseling.

## Discussion

This case describes an older patient with a history of increased PTH, several jaw lesions, and kidney cancer, all clinical manifestations of HPT-JT, which was diagnosed by genetic testing. The patient presented to our department with jaw tumors that were infected after removal of adjacent teeth. Jaw tumors, despite the syndrome nomenclature, occur in only one-third of cases [8]. Jaw tumors with histological diagnosis of ossifying fibroma, accompanied by increased PTH, can indicate initial clinical presentation of HPT-JT [9]. In this case, the histopathological diagnosis of ossifying fibroma was confirmed in the upper left maxilla. The clinical and radiological findings also supported the diagnosis of an ossifying fibroma in the lower right jaw. Multiple ossifying fibromas are exceedingly rare, and only 16 cases have been reported. The incidence of multiple ossifying fibromas in cases of known HPT-JT with jaw tumor is 33.3% [10].

Parathyroid disorders, of which PHPT is the most common, are reported to increase in incidence. In most cases, the PHPT is sporadic and caused by a single parathyroid adenoma. The heritable causes of PHPT comprise 10% to 15%, with the most common forms being multiple endocrine neoplasia type 1 (MEN1) and type 2 (MEN2). HPT-JT is much less common. MEN1 and MEN2 typically are multiglandular with MEN2 less consistently so than MEN1, while HPT-JT is a predominantly uniglandular disease [11].

HPT-JT is associated with a higher prevalence of PC, unlike MEN1 and MEN2 where parathyroid tumors generally are benign [8]. The CDC73 pathogenic variants with loss of parafibromin expression have been clearly coupled to parathyroid malignity [12]. In patients with HPT-JT, early parathyroidectomy because of the high risk of PC is indicated [13].

The international guidelines on evaluation and management of PHPT suggest genetic testing performed on patients with young-onset PHPT (age ≤30 years), multiglandular disease, family history of hypercalcemia or syndromic diseases (MEN1, MEN2, HPT-JT), or atypical parathyroid adenoma or PC to uncover and identify heritable origins of the disease [13]. If current guidelines had been available at the time of this patient's initial PHPT diagnosis, the HPT-JT could have been diagnosed many decades earlier. However, with the rarity of the syndrome and limited literature available, it would still have been a challenging diagnosis.

Early detection and diagnosis of HPT-JT via genetic testing can reduce sickness-related morbidity and mortality of patients due to increased surveillance and monitoring. There are no established guidelines for surveillance of known *CDC73* mutation carriers, but recommendations are multidisciplinary, including monitoring serum calcium and PTH for PHPT screening, panoramic x-ray imaging of the jaws at least every 5 years, monitoring for kidney lesions by periodic renal ultrasound, magnetic resonance scan or CT scan at least every 5 years, and, for females from reproductive age, regular gynecologic care including imaging studies [8].

## Learning Points

- HPT-JT is a rare syndrome with PHPT as a core feature, but it can manifest clinically with jaw tumors.
- Jaw tumors with a diagnosis of ossifying fibroma with concurrent increased PTH should undergo genetic testing for HPT-JT.
- PHPT in younger patients (age ≤30 years) prompts genetic testing for underlying heritable causes.
- There is a significant risk of PC in patients with HPT-JT.

## Contributors

All authors made individual contributions to authorship. P.S.J., P.F., A.N.N., and M.M. were involved in the diagnosis and management of the patient and manuscript submission. M.M. and P.S.J. were responsible for the patient's surgeries. K.A. was responsible for histopathological diagnosis and image. All authors reviewed and approved the final draft.

## Data Availability

Data sharing is not applicable to this article as no datasets were generated or analyzed during the current study.
